# Obstructive Sleep Apnoea and Atrial Fibrillation: Current Evidence and Emerging Research Directions

**DOI:** 10.31083/RCM50804

**Published:** 2026-08-05

**Authors:** Paweł Basiukiewicz, Shaoije Chen, Mark T. Mills, Piotr Futyma

**Affiliations:** ^1^Cardiac Arrhythmia Outpatient Unit, St. John Paul II Western Hospital, 05-825 Grodzisk Mazowiecki, Poland; ^2^Primula Clinics, Cardiology Outpatient Clinic and Sleep Apnoea Laboratory, 05-825 Grodzisk Mazowiecki, Poland; ^3^Department of Internal Medicine B (Cardiology, Angiology, Pneumology and Internal Intensive Care Medicine), German Centre for Cardiovascular Research (DZHK), University Medicine Greifswald, 17475 Greifswald, Germany; ^4^Rhythmology and Clinical Cardiac Electrophysiology, Klinik Internal Medicine B (Cardiology, Angiology, Pneumology and Internal Intensive Care Medicine), University Medicine Greifswald, 17475 Greifswald, Germany; ^5^Department of Cardiology, Sheffield Teaching Hospitals NHS Foundation Trust, S10 2JF Sheffield, UK; ^6^St. Joseph’s Heart Rhythm Center, 35-623 Rzeszów, Poland; ^7^Medical College, University of Rzeszów, 35-310 Rzeszów, Poland

**Keywords:** atrial fibrillation, obstructive sleep apnoea, pulmonary vein isolation, catheter ablation, obesity, arrhythmia

## Abstract

This review summarizes current knowledge on the coexistence of obstructive sleep apnoea and atrial fibrillation. A narrative review of the literature was conducted to identify relevant clinical and mechanistic studies. Obstructive sleep apnoea is highly prevalent among patients with atrial fibrillation and adversely affects rhythm-control outcomes. The interplay between these two conditions is of significant clinical importance, as obstructive sleep apnoea contributes to the development and progression of arrhythmias through multiple pathophysiological mechanisms: mechanical atrial stretch, immune activation and low-grade inflammation, autonomic dysregulation, oxidative stress, and systemic hypoxia. These mechanisms promote the initiation, maintenance, and treatment resistance of atrial fibrillation. Multimodal management strategies aimed at this harmful interplay may improve outcomes in patients with atrial fibrillation and include lifestyle and dietary interventions, pharmacometabolic therapies, particularly glucagon-like peptide-1 receptor agonists, continuous positive airway pressure therapy, and, in selected cases, catheter ablation strategies, potentially extended to the ganglionated plexi. Collectively, these approaches may contribute to the restoration and maintenance of sinus rhythm.

## 1. Introduction

Atrial fibrillation (AF) and obstructive sleep apnoea (OSA) are diseases with high global prevalence and growing clinical impact. Their strong association with modifiable lifestyle factors, such as lack of physical activity and an unhealthy diet leading to obesity, makes them directly lifestyle-related diseases. While obesity and its metabolic sequelae represent key drivers of contemporary cardiovascular disease [1], AF and OSA are not explicitly listed in the WHO report. Both conditions do, however, fulfil the definition of non-communicable diseases and are strongly influenced by lifestyle and environmental factors [2,3].

AF and OSA often coexist and are interconnected through multiple mechanisms. The relationship between the two conditions is bidirectional: the presence and progression of AF may adversely affect sleep quality, while OSA contributes to AF initiation, progression, and treatment resistance. Obesity increases the risk and worsens the course of both conditions [4,5,6,7,8,9,10,11]. In conclusion, we propose establishing the concept of Metabolic Arrhythmia–Respiratory Syndrome (MARS) as a comprehensive term capturing this interconnected disease entity.

This narrative review proposes that AF and OSA represent intersecting manifestations of a shared cardiometabolic–autonomic disease state, in which factors such as obesity, intermittent hypoxia, autonomic imbalance, and atrial stretch jointly drive arrhythmogenesis and create therapeutic opportunities beyond rhythm control alone. For this review, we searched PubMed using the following query: “((sleep AND apnoea) OR OSA OR OSAS) AND (obesity OR (atrial AND fibrillation OR AF OR AFib))”. We also used the OpenEvidence platform to perform contextual searches for relevant publications. Priority was given to contemporary epidemiological studies, meta-analyses, randomized controlled trials, and mechanistic or interventional research relevant to the interaction between OSA and AF. The search was limited to English-language literature. The search and initial screening were performed by PB, with subsequent review by PF, MM, and SC.

## 2. Obstructive Sleep Apnoea as a Risk Factor for Atrial Fibrillation

OSA is widely recognized as a condition associated with a broad spectrum of cardiovascular diseases, particularly heart failure with preserved ejection fraction (HFpEF), hypertension, and AF [12,13,14]. It is clear that OSA requires more specific recommendations targeted at patients with cardiovascular disease [15]. Older meta-analyses (2018) report that the prevalence of OSA in patients with AF ranges from 21% to 72% [16]. In the most recent and comprehensive meta-analysis (2025), comprising 38 studies—including 7 randomized controlled trials—the prevalence of OSA among patients with AF showed substantial heterogeneity, ranging from 5% to 90%, with a pooled mean prevalence of approximately 60% [17]. This marked variability is largely explained by differences in diagnostic strategy rather than true biological heterogeneity. It should be emphasized that, in this review of 38 studies, eight studies did not use any objective technology to establish the diagnosis of OSA, relying solely on electronic medical records. In the remaining studies, the diagnosis was established using home sleep apnoea testing (HSAT), polysomnography (PSG) or data derived from implanted cardiac devices. Studies relying on record-based diagnoses consistently reported substantially lower OSA prevalence, suggesting significant under-recognition of OSA in routine AF care.

Another study by Chen et al. [18] has shown a linear relationship between OSA severity and AF burden. This association was accompanied by progressive dilatation of the cardiac chambers. Moderate OSA was associated with a 3.05-fold increase in the odds of AF, whereas severe OSA carried a 3.88-fold (odds ratio (OR) 3.881; 95% confidence interval (CI); 1.306–11.534) increase in the odds of AF [18]. Studies demonstrating heterogeneity in the prevalence of obstructive sleep apnoea in atrial fibrillation populations, and its dependence on the diagnostic method used, are listed in Table 1 [19,20,21,22,23,24,25,26,27].

**Table 1.** **OSA prevalence in populations with diagnosed AF**.

Study	Study design	Diagnostic method	OSA prevalence
Feng, 2019, J Clin Anesth [19]	Retrospective	Medical records analysis	6%
Providencia, 2019, J Am Heart Assoc [20]	Retrospective	Medical records analysis	7%
Gami, 2004, Circulation [21]	Prospective	Berlin questionnaire	49%
Stevenson, 2008, Eur Heart J. [22]	Prospective	Portable PSG	62% (AHI >15)
Shapira-Daniels, 2020, JACC Clin Electroph [23]	Prospective	HSAT	82.4%
Tanaka, 2021, Circ J. [24]	Retrospective	HSAT (WatchPAT)	88.6%
Mills, 2023, American Heart Journal [25]	Prospective	HSAT (WatchPAT)	84%
Jensen, 2023, Int J Cardiol Heart Vasc [26]	Prospective	HSAT (The NO system)	92%
Ben Halima, 2020, Tunis. Medicale [27]	Prospective	HSAT	90% (new diagnosis)

Reported prevalence varies markedly according to diagnostic modality, with studies using HSAT identifying substantially higher rates of previously unrecognized OSA. Abbreviations: HSAT, home sleep apnoea testing; OSA, obstructive sleep apnoea; AF, atrial fibrillation; PSG, polysomnography; AHI, apnoea-hypopnoea index.

## 3. Daytime Sleepiness

In 1991, the Epworth Sleepiness Scale was developed and has remained in widespread clinical use to this day [28]. A study by Albuquerque and colleagues [29] showed that 35% of the AF population had excessive daytime sleepiness, defined by an Epworth Sleepiness Scale (ESS) score ≥11, while in the same cohort, 81.4% of patients were diagnosed with OSA. The sensitivity of the ESS for detecting OSA was 32.2%, whereas the specificity was 54.5% [29]. Similarly, in a study by Kadhim et al. [30], an ESS ≥11 was reported in 11.9% of patients with AF and an apnoea-hypopnoea index (AHI) ≥5. It was poorly associated with the diagnosis of moderate-to-severe OSA, with a negative predictive value of 43.1% and a positive predictive value of 67.5%. The authors concluded that in patients with AF, the ESS does not correlate well with the severity of sleep-disordered breathing, nor does it adequately identify individuals at risk of having OSA. This may reflect an AF-specific phenotype characterized by altered symptom perception, autonomic adaptation, or reduced hypersomnolence despite significant nocturnal respiratory disturbance. Given these findings and considering that positive airway pressure (PAP) therapy is currently recommended primarily for patients with OSA who present with significant daytime sleepiness [31], the high prevalence of OSA detected in HSAT or PSG studies does not directly translate into eligibility for PAP therapy under current guideline frameworks.

## 4. Night-to-Night Variability of Obstructive Sleep Apnoea Severity

Night-to-night variability reflects substantial intra-individual fluctuation in OSA severity across consecutive sleep studies. As shown in a meta-analysis of 3250 participants by Roeder et al. [32], 41% of subjects with co-existing OSA and AF demonstrated a change in AHI of more than 10 events per hour between nights. Moreover, 49% of participants shifted to a different severity category (defined as mild– AHI >5, moderate– 15 < AHI < 30, and severe– AHI >30 events/hour) [32]. These findings indicate that clinically relevant OSA may be misclassified when diagnosis is based on a single-night assessment, with a systematic tendency toward underestimation of disease severity. This raises concerns about the diagnostic accuracy and reproducibility of commonly used assessment methods.

## 5. Mechanisms Involved in Obstructive Sleep Apnoea-Driven Atrial Fibrillation

**Fig. 1.**
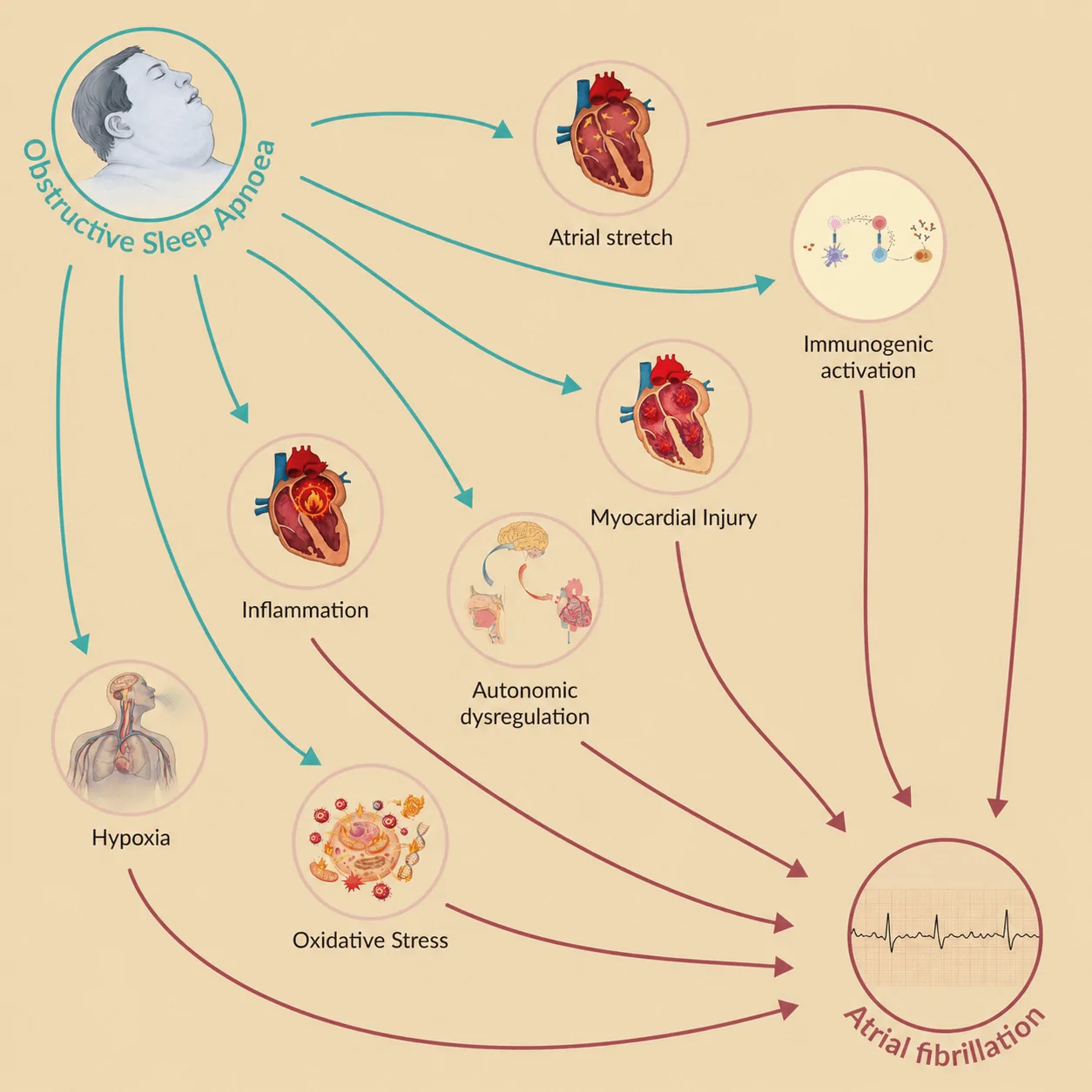
**Schematic overview of the pathophysiological mechanisms underlying atrial fibrillation associated with obstructive sleep apnoea**.

Multiple mechanistic pathways have been proposed to explain the role of OSA as a driver of AF, including microstructural myocardial injury, inflammation and immunogenic activation, autonomic dysregulation, intermittent hypoxia, oxidative stress, and repetitive mechanical stretch of the atrial myocardium [33,34]. A schematic overview of the pathophysiological mechanisms underlying atrial fibrillation associated with obstructive sleep apnoea is presented in Fig. 1.

### 5.1 Microstructural Myocardial Injury

Evidence of microstructural cardiac damage has been observed in patients with OSA, with detectable plasma troponin levels reported in 62–83.5% of cases, increasing to as high as 95% among cohorts with evidence of endothelial dysfunction [35,36]. In the PARAGON-HF subpopulation with concomitant OSA (approximately 12%), treated with sacubitril/valsartan for HFpEF, the HFpEF phenotype was more frequently associated with biventricular diastolic dysfunction worse absolute left ventricular (LV) global longitudinal strain (15% vs 16%), and worse absolute right ventricular (RV) free wall longitudinal strain (17% vs 19%). and myocardial hypertrophy (greater adjusted LV mass index (93 vs 87 g/m^2^)), independent of left ventricular ejection fraction [37]. These observations support the concept that OSA contributes to a diffuse myocardial disease process extending beyond the atria.

### 5.2 Atrial Injury and Fibrosis

Animal studies also provide mechanistic evidence of cardiac injury. A study in mice showed that chronic intermittent hypoxia was associated with increased atrial structural remodeling, including a significantly larger left atrial diameter, enhanced fibrosis, and elevated levels of TNF-α and IL-6 [38]. In rats exposed to intermittent hypoxia, there was marked atrial conduction slowing, increased atrial fibrous tissue content, left ventricular hypertrophy, dilation, diastolic dysfunction, and enhanced AF inducibility [39]. Atrial tissue analysis in patients with OSA revealed significantly greater interstitial fibrosis [40].

### 5.3 Inflammation. Role of Adipokines

In patients with coexisting OSA and AF, elevated concentrations of inflammatory and immunogenic biomarkers such as Galectin-3 (GAL), Pentraxin-3 (PTX3), Growth Differentiation Factor-15 (GDF-15), Matrix Metalloproteinase-9 (MMP-9), Vascular Cell Adhesion Molecule-1 (VCAM-1), and Angiopoietin-2 (ANGP2) have been observed. Higher PTX3 levels have been shown to be an independent predictor of AF recurrence and stroke risk following ablation [41]. In a cardiac surgery cohort, patients with OSA had a higher incidence of postoperative AF and significantly higher baseline levels of NT-proBNP, vWF, Gal-3, IL-6, and CRP [42]. An OSA rat model shows that OSA promotes M1 macrophage polarization, which contributes to atrial remodeling through the secretion of inflammatory cytokines, thereby increasing susceptibility to AF [40].

Recent data suggest that more than 95% of patients with HFpEF exhibit metabolic features of obesity. While only approximately 60% have a body mass index (BMI) >30 kg/m^2^, over 95% show a waist-to-height ratio (WHtR) >0.5. The underlying pathophysiological substrate of the HFpEF syndrome—including both structural abnormalities and symptom burden—appears to be driven by excessive secretion of domain III adipokines, a group of pro-inflammatory cytokines that promote myocardial fibrosis and hypertrophy [43]. Cardiac adipokines are also elevated in OSA. Among these, leptin and chemerin are found at increased concentrations in patients with coexisting AF and OSA and directly contribute to myocardial hypertrophy, fibrosis, and chronic low-grade systemic inflammation [44,45,46]. In the rat model, prolonged chronic intermittent hypoxia exposure (96 days) led to increased body weight, higher food intake, and reduced locomotor activity—despite significantly elevated circulating leptin levels. Chronic intermittent hypoxia induces central leptin resistance, driven by elevated leptin levels, disrupted receptor signaling, and downstream pathway inhibition [47]. In contrast to the rat model, *in vitro* studies in human adipocytes show that acute intermittent hypoxia increases leptin secretion, whereas prolonged intermittent hypoxia (7 days) decreases leptin secretion [48]. Moreover, a study in rats by Breuillard (2023) [49] demonstrated that chronic intermittent hypoxia reduced food intake and resulted in lower body mass after 14 days of observation. These findings from animal and *in vitro* studies do not provide a consistent explanation of the effects of chronic intermittent hypoxia on leptin concentrations. Moreover, in the clinical setting, neither AF catheter ablation nor PAP therapy has been shown to consistently reduce circulating leptin concentrations, suggesting that upstream metabolic and inflammatory drivers may persist despite rhythm control or OSA-targeted therapies [50,51,52].

### 5.4 Autonomic Imbalance

The cardiac autonomic nervous system represents one of the central integrative hubs linking the pathophysiology of OSA and AF. It comprises the extrinsic autonomic nervous system, which includes neural pathways projecting from the central nervous system to the heart, and the intrinsic cardiac nervous system, formed by clusters of autonomic ganglia embedded in the fat pads surrounding the pulmonary veins. Sympathetic nerve fibers forming the extrinsic cardiac nervous system originate predominantly from the superior cervical ganglia and the stellate ganglia [53,54]. In the course of OSA, damage to the autonomic nervous system (particularly small-fiber neuropathy) occurs more frequently, and its severity appears proportional to OSA severity [55]. Episodes of oxygen desaturation, as well as increased heart rate variability indicative of autonomic dysfunction, are associated with a higher incidence of AF in patients with OSA [56,57]. In the study by Chen et al. [18], in patients with severe OSA, non-sustained ventricular tachycardia was associated with heart rate variability (HRV) parameters (the low-frequency to high-frequency ratio (LF/HF ratio) as an indicator of autonomic imbalance), along with echocardiographic markers linked to AF occurrence. Healthy individuals exposed to 10 days of intermittent hypoxia, modeled on oxygen saturation patterns observed in OSA, show increased sympathetic nerve activity [58]. Exposure of healthy subjects to hypobaric hypoxia for 4 weeks, simulating an altitude of 5260 m, leads to increased sympathetic nerve activity (SNA), which persists for up to 3 days after return to sea level [59].

The surge in sympathetic activity overlapping with a concomitant increase in vagal tone at the end of apnoea episodes is associated with a heightened risk of AF initiation and stroke [60]. In experimental OSA models, a lower AF inducibility threshold and a shortened right atrial refractory period have been observed. Experimental modulation of autonomic ganglia, particularly when combined with low-voltage vagal stimulation, reduces arrhythmic susceptibility [61,62,63,64]. Despite these observations, the precise contribution of autonomic dysregulation to OSA-mediated AF remains incompletely defined. Changes in intrathoracic pressure during obstructive events (Mueller maneuver) result in cardiac chamber distension, atrial stretch, and sympathetic activation. Repetitive pressure swings shorten atrial refractory periods and, over time, promote mechanical remodeling and myocardial fibrosis [16,34].

### 5.5 Oxidative Stress

Oxidative stress arises from an imbalance between reactive oxygen species (ROS) generation and antioxidant defenses, leading to oxidative damage of cellular macromolecules and tissue injury [65]. Multiple animal and basic studies have shown that oxidative stress plays a crucial role in tissue damage. Evidence indicates that intermittent negative intrathoracic pressure and chronic intermittent hypoxia cause a ROS imbalance, leading to altered gene expression and subsequent cellular and tissue injury that may be associated with longer inducible AF episodes [66,67,68,69,70,71].

### 5.6 Atrial Stretch

Inspiratory efforts against an occluded upper airway generate markedly negative intrathoracic pressure, lowering pericardial and pleural pressures and thereby augmenting transmural pressure across the cardiac walls. This effect is most pronounced in the thin-walled atria. During obstructive apnoea, intrathoracic pressure may transiently decrease to levels as low as −60 mmHg [16]. Acute axial stretch of isolated mouse atrial myocytes increases diastolic sarcoplasmic reticulum Ca^2+^ leak, which is mechanistically linked to increased susceptibility to atrial fibrillation in pressure-overloaded hearts [72]. Mechanical stretch of isolated atrial tissue may increase susceptibility to alternans, which may contribute to the promotion of atrial fibrillation [73]. In a rat model in which atrial enlargement was induced by balloon inflation in both atria, the distended atria showed increased susceptibility to atrial fibrillation [74]. Conversely, experimental unloading of left ventricular and left atrial pressures is associated with reduced susceptibility to arrhythmias [75].

## 6. Effect of Positive Airway Pressure Therapy on Atrial Fibrillation Recurrence

Some available evidence suggests a beneficial effect of PAP therapy on AF burden and recurrence in cohorts with coexisting OSA and AF. However, these findings are derived predominantly from observational trials and meta-analyses heavily weighted toward non-randomized data, which limits the strength of causal inference. In the meta-analysis by Trongtorsak et al. [76], the use of continuous positive airway pressure (CPAP) in patients with OSA was associated with significantly lower AF recurrence after pulmonary vein isolation (PVI) (OR = 0.36, 95% CI 0.25–0.53, *p* < 0.001); however, among 1487 participants included across eight studies, only 83 were from a single randomized controlled trial (Hunt et al. [77]), whereas the remaining 1404 were derived from seven observational studies. Similarly, in the meta-analysis by Itaya et al. [78], in the pooled analysis, AF recurrence (OR 0.37; 95% CI 0.23–0.58; *p* < 0.01) was significantly lower in patients treated with CPAP compared with no CPAP; however, among 1536 participants from 11 studies, only 83 were from a randomized controlled trial (Hunt et al. [77]), with the remainder originating from observational studies. In the meta-analysis by Li et al. [79], CPAP therapy was associated with a reduced risk of AF recurrence or progression (24.8% vs 40.5%, risk ratio [RR] = 0.70, 95% CI = 0.57–0.85, *p* = 0.035), but only one randomized controlled trial with 34 participants was included, compared with eight observational studies comprising 14,812 participants. Likewise, in the analysis by Li Feng et al. [80], CPAP therapy was associated with lower AF incidence (OR 0.58, *p* < 0.05), but only one randomized controlled trial with 83 participants (Hunt et al. [77]) was included alongside seven observational studies with 1231 participants. Taken together, this predominance of observational data substantially affects the overall quality of evidence and limits causal inference [76,78,79,80,81]. Conversely, randomized controlled trials have not demonstrated a significant effect of PAP therapy on AF recurrence or burden; however, these studies were substantially underpowered or otherwise methodologically limited. Across four randomized controlled trials (Traaen et al., 2021 [82]; Hunt et al., 2022 [77]; Nalliah et al., 2022 [83]; Caples et al., 2019 [84]), the number of patients included in follow-up was limited, comprising 158 patients (25% unable to tolerate PAP therapy), 83 patients, 24 patients, and 25 patients, respectively [77,82,83,84]. Conversely, the neutral-outcome randomized controlled trial (RCT) by McEvoy et al. [85] (SAVE trial) was adequately powered and of sufficient duration; however, mean PAP adherence was only 3.3 h/night, a level generally regarded as insufficient for therapeutic efficacy. This apparent discrepancy may reflect differences in patient selection, OSA phenotyping, PAP adherence, and endpoint definition across studies. Nevertheless, PAP therapy may exert favorable effects on cardiac structure, including partial normalization of atrial dimensions and reversal of pressure-related remodeling [86].

## 7. Obesity and Obstructive Sleep Apnoea in Patients With Atrial Fibrillation

Obesity represents an independent risk factor for AF, conferring a risk that extends beyond the direct proarrhythmic effects of obesity-related comorbidities such as hypertension and diabetes [4,5]. The diagnosis of AF is more likely to be established in patients with a BMI above 30 kg/m^2^ than in those with a BMI below this threshold [6,7]. There is a 29% (OR: 1.29, 95% CI: 1.23 to 1.36) and a 19% (OR: 1.19, 95% CI: 1.13 to 1.26) greater excess risks of incident AF for every 5-unit BMI increase in cohort and case-control studies, respectively [8]. Moreover, elevated abdominal adiposity confers a significantly increased risk of AF even in individuals without obesity as defined by BMI >30 kg/m^2^, highlighting the limitations of BMI as a sole risk marker [87]. BMI is best used as a marker of cardiometabolic risk at the population level. At the individual level, excess adiposity is more accurately characterized using direct measures of central fat distribution, such as waist-to-hip ratio, WHtR, or waist circumference [88]. WHtR is considered one of the most robust indicators of visceral obesity and demonstrates strong correlations with metabolic disturbances such as type 2 diabetes, hypertension, cardiovascular diseases, dyslipidemia, and insulin resistance [9,10,89,90]. The relationship between OSA and obesity is bidirectional—as BMI and WHtR increase, both the likelihood and the severity of OSA rise. Conversely, the worsening course of OSA contributes to hormonal and behavioral changes that facilitate weight gain, establishing a self-reinforcing pathological cycle. A 10% increase in BMI has been associated with up to a six-fold higher risk of moderate-to-severe OSA [11].

Sleep deprivation in OSA activates multiple endocrine mechanisms that favor weight gain. Growth hormone, which is secreted predominantly during slow-wave sleep under physiological conditions, is reduced in patients with OSA due to fragmentation and loss of deep sleep, leading to impaired lipolysis, fat accumulation and reduced musculoskeletal regeneration. Ghrelin, an appetite-stimulating hormone, is released predominantly during wakefulness and is upregulated in sleep deprivation, promoting increased caloric intake. In contrast, leptin—an appetite-suppressing hormone that also stimulates the respiratory center—is secreted in elevated concentrations during apneic episodes, resulting in leptin resistance and attenuation of its anorexigenic effects [91,92,93,94,95]. Moreover, persistent sleep fragmentation enhances systemic pro-inflammatory activity, contributing not only to the promotion of arrhythmias but also to the development of obesity and cardiovascular complications [96,97,98,99,100]. Conversely, sleep extension in overweight individuals has been shown to lead to a reduction in calorie intake and a negative energy balance [101].

## 8. Role of Obesity Management, Lifestyle Modification and Incretin-Based Therapies in Obstructive Sleep Apnoea and Atrial Fibrillation

**Fig. 2.**
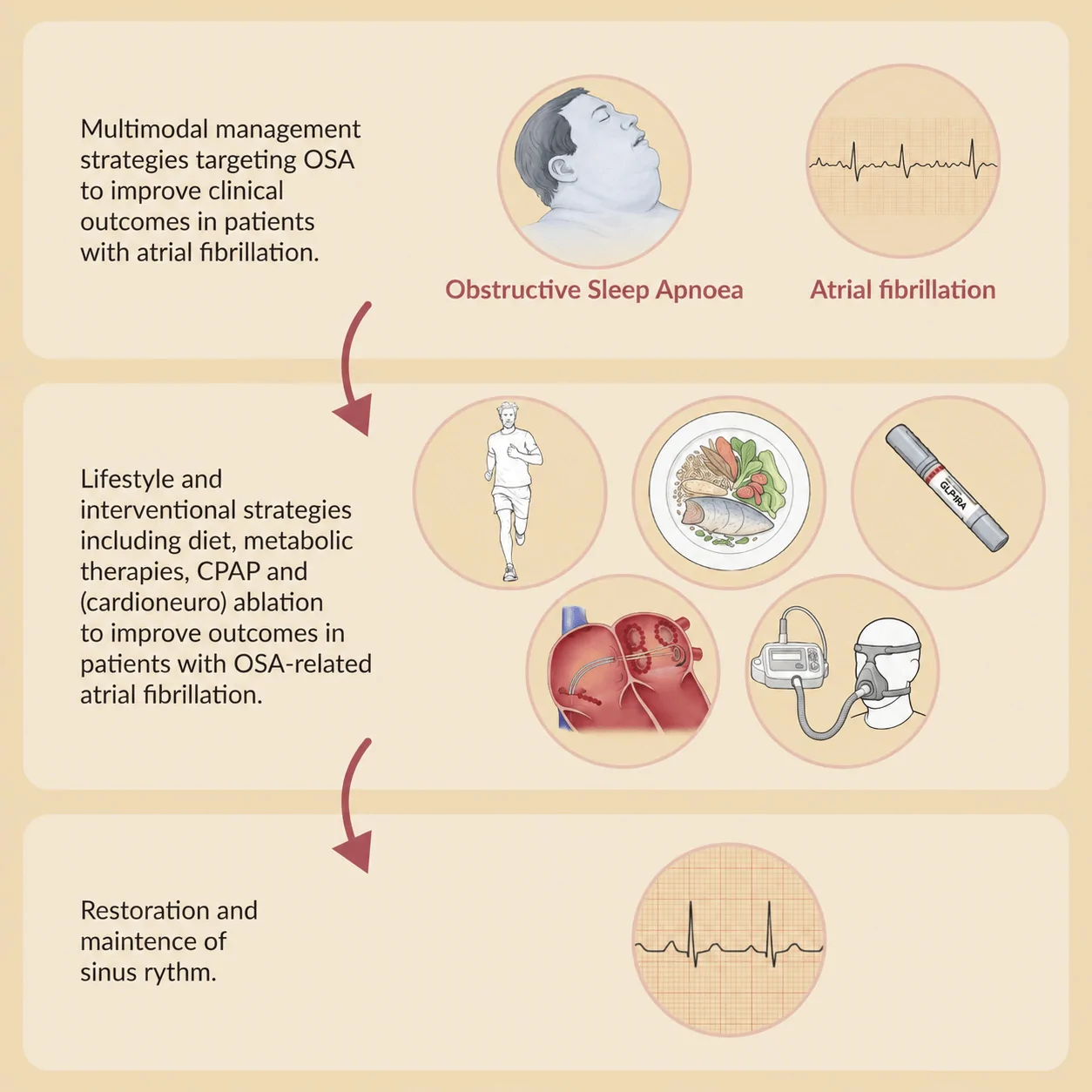
**Schematic overview of comprehensive risk factor management**.

This section focuses on the role of obesity, lifestyle factors, and emerging metabolic pharmacotherapies, including incretin-based agents, in the management of AF and OSA. We first discuss the impact of lifestyle-related risk factors and metabolic interventions on AF progression. Subsequently, we examine the effects of obesity management, lifestyle modification, and metabolic therapies on the course of OSA. Together, these data highlight that shared risk factors, and common intervention strategies may simultaneously improve outcomes in both conditions. A schematic overview of comprehensive risk factor management is presented in Fig. 2.

### 8.1 Improvement of Arrhythmia Management Through Targeting Lifestyle-Related Risk Factors

Lifestyle management, including weight loss and regular physical activity, plays a central role in AF management. However, as demonstrated by a 2025 survey conducted by the European Heart Rhythm Association (EHRA), there remains suboptimal awareness and inconsistent implementation of structured lifestyle interventions among clinicians involved in arrhythmia care [102].

Among modifiable risk factors, body weight and the management of coexisting metabolic abnormalities exert particularly strong influences on AF outcomes. Dietary intervention has been shown to improve ablation outcomes in patients with AF 1% change in weight that occurred during the year before ablation independently predicted freedom from AF through 15-months (adjusted odds ratio (aOR) = 1.17, 95% CI: 1.11–1.23) [103]. Addressing lifestyle-related risk factors through structured, specialist-led behavioral modification programmes improves long-term rhythm control and treatment durability [104,105]. Weight loss achieved prior to AF catheter ablation is associated with improved AF-free survival, an effect observed in both obese and non-obese patients [103].

Comprehensive management of comorbidities, including structured lifestyle interventions targeting smoking cessation, alcohol reduction, and weight reduction, was linked to a twofold reduction (RR 0.49, 95% CI 0.30–0.78, *p* = 0.004) in AF recurrence within 12 months following PVI in the POP AF randomized controlled trial [105]. Further evidence supporting comprehensive lifestyle intervention was provided by the ARREST-AF trial, in which Pathak et al. (2014) [106] demonstrated improved outcomes – risk factor modification resulted in greater reductions in weight (*p* = 0.002) blood pressure (*p* = 0.006), better glycemic control (*p* = 0.001) and improved lipid profiles (*p* = 0.01). At follow-up, AF frequency, duration, symptoms, and symptom severity decreased more in the risk factor management group compared with the control group (all *p* < 0.001) [106]. These findings were subsequently confirmed in a randomized controlled trial conducted by the same research group, risk of recurrent arrhythmia over 12 months was 0.53 (95% CI, 0.32–0.89) for the risk factor management vs usual care [107].

Similarly, the LEGACY study demonstrated that achieving at least 10% sustained weight loss was associated with significant improvement in metabolic profile and a marked reduction in arrhythmia recurrence (Weight loss ≥10% resulted in a 6-fold (95% CI: 3.4 to 10.3; *p* < 0.001 of arrhytmia free survival)). Importantly, weight fluctuation, whether gain–loss or loss–gain, was associated with a twofold increase in AF recurrence [108].

In another study, Pathak et al. [109] showed that an improvement of more than 2 metabolic equivalents (METs) in cardiorespiratory fitness provided independent benefits for arrhythmia-free survival, exceeding the protective effect of weight loss alone by approximately 12%. Although the SORT-AF trial did not demonstrate a significant overall reduction in AF recurrence with weight loss, subgroup analyses suggested a reduction in arrhythmia burden among patients with persistent AF. Similarly, Mohanty et al. [110] reported substantial improvement in quality of life associated with weight loss, despite no significant change in arrhythmia recurrence [111].

Recently published results from the PRAGUE-25 randomized clinical trial compared AF ablation plus usual care with lifestyle modification combined with antiarrhythmic drug therapy. While patients in the lifestyle modification plus antiarrhythmic drug group achieved greater weight loss, improved glycemic control (HbA1c), and higher peak oxygen consumption (VO_2_), a lower rate of arrhythmia recurrence (defined as ≥30 seconds and detected by an implantable loop recorder) was observed in the ablation group over two years of follow-up [112].

It should be emphasized that across several of these studies, including the ARREST-AF prospective observational study, the ARREST-AF randomized controlled trial, and PRAGUE-25, obstructive sleep apnoea accounted for approximately 35–45% of the modifiable risk-factor burden. Its management was associated with reduced atrial fibrillation recurrence, alongside broader health benefits such as lower blood pressure, substantial weight loss, and metabolic improvement [106,107,112]. Overall, these findings highlight the complementary rather than competing roles of interventional rhythm control and sustained lifestyle modification in AF management.

### 8.2 Improving Arrhythmia Management Through Metabolic Pharmacotherapy

Given the well-documented cardiometabolic benefits of glucagon-like peptide-1 receptor agonists (GLP-1RA), it is reasonable to anticipate overall clinical improvement in patients with AF and obesity. Beyond their metabolic effects, there is a plausible mechanistic rationale for direct antiarrhythmic properties of GLP-1RA. In an experimental study, GLP-1RA administration reduced spontaneous pulmonary vein activity in a concentration-dependent manner, suggesting a potential antiarrhythmic effect mediated through regulation of protein kinase A (PKA), Ca^2+^/calmodulin-dependent protein kinase II (CaMKII), and the sodium–calcium exchanger (NCX), as well as modulation of intracellular Ca^2+^ homeostasis [113].

Emerging evidence suggests that GLP-1RA therapy may be associated with a reduction in AF burden among individuals with obesity. A meta-analysis of 26 randomized controlled trials reported a lower incidence of AF among patients treated with semaglutide. Therefore, GLP-1RA and GLP-1/GIP agonists may represent promising pharmacotherapeutic strategies for targeting AF through obesity management [114]. Similarly, beneficial effects on AF-related outcomes—including reduced use of antiarrhythmic drugs (AADs), lower need for direct current cardioversion (DCC), fewer repeat AF ablations, and reduced mortality—have been reported in propensity score–matched cohorts [115]. These observations are supported by a meta-analysis of randomized controlled trials including patients with HFpEF and heart failure with reduced ejection fraction (HFrEF) and concomitant AF, which demonstrated a reduction in AF incidence associated with GLP-1RA therapy [116].

In a prospective cohort study of patients with a cardiac resynchronization therapy defibrillator (CRT-D) and diabetes, combined therapy with GLP-1RA and sodium-glucose cotransporter-2 inhibitor (SGLT2i) was associated with lower AF recurrence compared with either monotherapy. However, patients receiving combination therapy exhibited worse glycemic control [117]. Another meta-analysis of 12,651 patients at high cardiovascular risk drawn from randomized controlled trials demonstrated a 42% reduction in AF incidence compared with placebo [118]. In a matched cohort of 14,566 patients receiving either GLP-1RA or DPP-4i therapy, GLP-1RA use was associated with a lower risk of AF over nearly four years of follow-up [119].

A recently published prospective observational study by Guo et al. [120] reported a 32% reduction in AF recurrence after ablation among patients treated with semaglutide (BMI >24 kg/m^2^). Conversely, in a similar retrospective comparison of GLP-1RA vs. DPP-4i, no difference in AF incidence was observed. However, when the analysis was restricted to patients who achieved meaningful weight loss while receiving GLP-1RA therapy, an antiarrhythmic effect emerged, suggesting that weight reduction and mitigation of adipotoxicity may represent the dominant mechanism underlying AF risk reduction [121]. In contrast, Satti et al. (2024) [122], in a propensity score–matched cohort of patients who received GLP-1RA therapy during the pre-ablation period, showed no beneficial effect of GLP-1RA use on AF recurrence, heart failure hospitalizations, or mortality. Moreover, in 56 RCTs involving approximately 80,000 participants, Wu et al. [123] also did not demonstrate a beneficial effect on AF incidence. Selected studies investigating the effects of GLP-1RA on AF burden are summarized in Table 2 (Ref. [114,115,116,117,118,119,120,121,122,123]).

**Table 2.** **Effects of GLP-1RA on AF burden and outcomes: review of selected studies**.

Study	Type	Year	Outcome
Cesaro et al., Eur J Prev Cardiol [114]	Systematic review	2025	↓ AF burden. (OR 0.83, 95% CI 0.7–0.98)
Patel et al., JCE [115]	Cohort study	2025	↓DCC, ↓AF recurrence, ↓re-do procedures; ↓ mortality. (HR 0.72 [95% CI 0.65–0.80])
Ravikulan et al., Eur J Heart Fail [116]	Meta-analysis	2025	↓AF in HF patients. (RR 0.54; 95% CI 0.36–0.81)
Sardu et al., Diab res Clin Practice [117]	Prospective, observational	2025	↓ amiodarone use (AF not mentioned). CRTD cohort. (HR 0.747; *p* = 0.019)
Saglietto et al., Eur J Clin Invest [118]	Meta-analysis	2024	↓ AF incidence by 42% (RR 0.58; 95% CI 0.4–0.85)
Xu et al., J Gen Intern Med [119]	Cohort study	2024	↓AF in GLP-1RA vs DPP-4i. (HR 0.82; 95% CI 0.7–0.96)
Khalil et al., Am J Cardiovasc Drugs [121]	Cohort study, propensity score matched	2025	↓ AF in GLP-1RA vs DPP-4i (only weight-loss group). (HR 0.83; 95% CI 0.78–0.98)
Guo et al. Circ Arrh Electr [120]	Prospective, observational	2025	↓ AF in semaglutide by 32% (HR 0.68; 95% CI 0.49–0.95)
Satti et al., JACC [122]	Cohort study, prop. Score matched	2024	No effect on AF
Wu et al., BMC diab et metabol [123]	Systematic review	2022	No effect on AF

Abbreviations: AF, atrial fibrillation; AAD, antiarrhythmic drugs; CRT-D, cardiac resynchronization therapy defibrillator; DCC, direct current cardioversion; HF, heart failure; GLP-1RA, glucagon-like peptide-1 receptor agonist; DPP-4i, dipeptidyl peptidase-4 inhibitor; HR, hazard ratio; RR, risk ratio; CI, confidence interval.

### 8.3 Improvement of Obstructive Sleep Apnoea Management Through Obesity and Lifestyle Interventions

Lifestyle intervention, including optimization of sleep hygiene, nutritional behavior, and regular physical activity, plays a central role in the management of OSA. Carneiro-Barrera et al. [124,125,126], in an open-label randomized clinical trial, demonstrated marked improvements in the lifestyle-intervention arm compared with usual care in OSA participants. Almost two-thirds of participants no longer required PAP after six months, and approximately 30% achieved complete remission of OSA. Additional benefits included significant reductions in body weight and improvements in body composition and quality of life. These effects were accompanied by improvements in cardiorespiratory fitness, self-reported physical fitness, as well as in daily functioning and psychiatric symptoms after eight weeks of intervention [124,125,126].

Comparable results were obtained in the MIMOSA trial (Georgoulis et al. [127]), in which participants with OSA were allocated to standard care, a Mediterranean diet intervention, or a Mediterranean lifestyle group. Participants receiving dietary or lifestyle interventions exhibited lower AHI, reduced daytime sleepiness, and longer rapid eye movement sleep compared with standard care [127]. Importantly, even modest weight loss (<5%) was sufficient to improve sleep-related parameters, whereas a reduction of more than 10% was required to reduce the prevalence of severe OSA (OR (95% CI) 0.33 (0.14, 0.81), *p* = 0.015) [128].

The importance of weight loss was further highlighted in a study by Fredheim et al. [129], in which participants with morbid obesity and OSA were assigned to either a lifestyle management or gastric bypass. The greatest weight loss occurred in the bariatric surgery group, with OSA remission achieved in 66% of participants. Notably, improvements in OSA were observed in both treatment arms and were directly proportional to the magnitude of weight loss [129]. Another clinical trial is currently underway in participants with a BMI of 30–45 and an established diagnosis of OSA. The study aims to determine whether self-directed lifestyle modification, when added to standard management, is sufficient to improve sleep quality and support weight-loss efforts. To date, no results are available, as the trial is still ongoing [130].

Conversely, short-term dietary interventions have produced inconsistent results. In a study by Schiavo et al. [131] evaluating a low-calorie ketogenic diet, no improvement in sleep quality was observed after four weeks, despite a reduction in C–reactive protein concentrations. Similarly, in a study by Cienfuegos et al. [132], time-restricted feeding did not lead to improvements in sleep quality despite significant weight loss after eight weeks of intervention. These neutral findings may be attributable to limited intervention duration and small sample sizes rather than a lack of biological effect.

In conclusion, the current evidence strongly supports lifestyle-based weight-loss interventions as a cornerstone of OSA management in patients with obesity, with benefits extending beyond sleep-related outcomes to broader cardiometabolic and functional health improvements.

### 8.4 Improvement of Obstructive Sleep Apnoea Management Through Metabolic Pharmacotherapy

Pharmacotherapies targeting the incretin axis are receiving increasing attention as potential disease-modifying treatments for OSA in individuals with obesity. Through their potent effects on body weight, fat distribution, and metabolic inflammation, GLP-1RA may indirectly improve the course of OSA. Malhotra et al. [133], in the SURMOUNT-OSA trial, demonstrated that tirzepatide (a dual GLP-1/GIP receptor agonist) significantly improved OSA severity by reducing body mass index and lowering AHI compared with placebo. Similar results were reported by Blackman et al. [134] in the SCALE Sleep Apnoea trial, in which liraglutide produced greater reductions in systolic blood pressure, HbA1c, and AHI than placebo. Jiang et al. [135], in an RCT involving patients with diabetes and OSA, demonstrated significant reductions in BMI, systolic blood pressure, and AHI compared with the control group [125]. These findings have been demonstrated in randomized controlled trials, which consistently showed significant reductions in AHI with liraglutide and tirzepatide treatment [126,127]. Taken together, current randomized and observational evidence supports consideration of GLP-1RA as adjunctive, disease-modifying therapy for OSA in carefully selected patients with obesity, particularly when lifestyle intervention alone is insufficient. Table 3 (Ref. [133,134,135,136,137]) summarizes OSA outcomes in patients receiving GLP-1RA.

**Table 3.** **Effects of GLP-1RA on OSA outcomes: review of selected publications**.

Author	Study type	Year	Outcome
Malhotra et al, NEJM [133]	RCT, tirzepatyd	2024	Improvement in AHI (95% CI, –25.8/h to –14.2/h) (*p* < 0.001)
Yang, et al., J Tran Med [137]	Meta-analysis	2025	Improvement in AHI in DM (MD = –5.68/h, 95% CI [–7.97/h, –3.38/h], *p* < 0.00001)
Jiang et al, Sleep Breath. [135]	RCT, liraglutide	2023	Improvement in AHI, BMI, sBP (AHI 26.1/h ± 7.1/h for liraglutide vs 31.6/h ± 6.9/h for placebo, *p* < 0.05).
Bardoczi et al., Sleep Rev Med. [136]	Meta-analysis	2025	Improvement in AHI (MD –11.61/h (95% CI: –22.91/h to –0.31/h, *p* = 0.046))
Blackman et al, Int J Obes. [134]	RCT, liraglutide	2016	Improvement in AHI (–12.2/h for liraglutide vs –6.1/h for placebo, estimated treatment difference –6.1/h (95% CI, –11 to –1.2, *p* = 0.015))

Abbreviations: RCT, randomized controlled trial; AHI, apnoea hypopnea index; BMI, body mass index.

## 9. Atrial Fibrillation Ablation in Patients With Obstructive Sleep Apnoea

### Limited Efficacy of Atrial Fibrillation Ablation

In some studies, the presence of OSA has been associated with reduced efficacy of AF ablation and has been identified as an independent predictor of post-procedural AF recurrence [138,139]. Undiagnosed OSA may increase the probability of AF recurrence almost twofold (51% versus 31%, *p* = 0.04) [140]. Moreover, shorter subjective sleep duration, longer objective sleep duration (likely reflecting poorer sleep quality), and prolonged nocturnal intermittent hypoxia have all been identified as predictors of AF recurrence following ablation [141].

In meta-analyses by Congrete et al. [142], Schukla et al. [143], as well as in the study by Fein et al. [144], OSA was associated with an increased risk of AF recurrence after catheter ablation, and, moreover, PAP therapy was shown to reduce the likelihood of recurrence [145].

According to a large retrospective cohort study by Bidaoui et al. [146], AF catheter ablation in patients with coexisting OSA was associated with improved cardiovascular outcomes, reflected by a reduction in major adverse cardiovascular events (MACE); (hazard ratio (HR) 0.596, 95% CI: 0.51–0.697), as well as reduced all-cause mortality (HR 0.264, 95% CI: 0.208–0.335). These findings suggest that effective rhythm-control strategies may partially mitigate the adverse cardiovascular consequences associated with OSA [146].

Conversely, not all studies have demonstrated a consistent association between OSA and AF ablation outcomes. In a prospective study by Hojo et al. [147], no effect of OSA on AF recurrence after ablation was observed. However, patients underwent not only PVI but also additional ablation at non–pulmonary vein foci, which may have influenced the results. Similarly, in a study by Lee et al. [148], AF recurrence did not correlate with OSA severity; however, ablation strategies extended beyond PVI to include complex fractionated atrial electrogram (CFAE) ablation and additional non–pulmonary vein targets at the operator’s discretion. Padeletti et al. [149] likewise reported no effect of OSA diagnosed using the Berlin Questionnaire on AF recurrence after ablation.

Targeted screening and intervention for obstructive sleep apnoea were integral components of studies evaluating structured risk-factor management strategies, as discussed in the section entitled “Improvement of arrhythmia management through targeting lifestyle-related risk factors”.

In summary, although data on the impact of OSA on AF ablation outcomes remain heterogeneous, the preponderance of evidence suggests that untreated OSA adversely affects rhythm-control success. Comprehensive risk-factor management, including OSA identification and treatment, improves clinical outcomes, while AF ablation remains effective in improving quality of life and may confer broader cardiovascular benefit irrespective of OSA status [150].

## 10. Directions for Future Research in Patients With Obstructive Sleep Apnoea Undergoing Pulmonary Vein Isolation for Atrial Fibrillation

As discussed above, small-fiber neuropathy develops in the course of OSA, reflecting its deleterious effects on the autonomic nervous system [55]. Exaggerated heart rate variability and nocturnal desaturations reflect surges in sympathetic and parasympathetic activity, which likely play an important role in arrhythmogenesis [56,57]. The cardiac autonomic nervous system is increasingly recognized as a key modulator of AF initiation and maintenance and may represent a therapeutic target in patients with coexisting AF and OSA [151].

Experimental studies support this hypothesis. Denervation of the right anterior ganglionated plexus has been shown to abolish apnoea-induced electrophysiological responses and reduce AF inducibility [64]. Similarly, ablation of the left superior ganglionated plexus has been associated with suppression of OSA-related AF [152]. When comparing AF ablation technologies, pulsed field ablation appears to induce less autonomic neural injury than thermal methods [153]. This relative autonomic sparing may have important implications for arrhythmia recurrence and symptom burden in patients with AF and coexisting OSA. Collectively, these observations support future investigation into the role of PVI combined with partial or targeted cardioneuroablation or alternative neuromodulatory strategies in this population.

## 11. Metabolic Arrhythmia–Respiratory Syndrome (MARS): Integrative Role of Obesity in OSA and AF

Given that central (visceral) obesity is a common feature of both AF and OSA, and that these conditions share overlapping pathophysiological mechanisms—including metabolic, inflammatory, autonomic, and structural pathways—interventions targeting obesity are likely to improve both disorders. We therefore propose the concept of Metabolic Arrhythmia–Respiratory-Syndrome as a comprehensive term describing this interconnected disease entity. Identifying such a clinical syndrome would facilitate targeted treatment—primarily through weight reduction—while preserving the important role of interventions such as ablation and CPAP for patients presenting with symptoms of AF or OSA.

## 12. Gaps in Knowledge

Despite growing interest in the interaction between OSA and AF, significant knowledge gaps remain. The majority of studies evaluating the impact of PAP therapy on AF outcomes in patients with coexisting OSA are observational, limiting causal inference. As a result, the true magnitude of benefit conferred by PAP therapy on AF recurrence and long-term outcomes remains uncertain, highlighting the need for adequately powered randomized controlled trials in this field. Furthermore, there are insufficient data examining the influence of AF burden or severity on the course and progression of OSA, representing an important but underexplored bidirectional relationship. Additionally, while emerging evidence implicates autonomic imbalance as a shared pathophysiological mechanism underlying both AF and OSA, robust evidence demonstrating that cardioneuromodulation effects achieved during PVI improve outcomes in this patient population is still lacking. Consequently, this remains a broad and largely unexplored field for future research.

## 13. Conclusions

OSA and AF are interconnected by a complex network of shared metabolic, autonomic, and humoral pathophysiological mechanisms. While OSA is well established as a contributor to AF development and recurrence, the extent to which AF influences the severity or clinical expression of OSA remains unclear and insufficiently studied. Given that central obesity is a common feature of both AF and OSA, and that these three conditions share overlapping pathophysiological mechanisms, interventions targeting obesity represent a rational and potentially high-impact therapeutic strategy. Weight reduction, implemented alongside established therapies such as catheter ablation and PAP therapy in appropriately selected symptomatic patients, may lead to improvements in rhythm control, symptom burden, and overall cardiometabolic health within a holistic treatment framework.

## Abbreviations

AAD, Antiarrhythmic Drugs; AHI, Apnoea–Hypopnea Index; AF, Atrial Fibrillation; ANGP2, Angiopoietin-2; BMI, Body Mass Index; CFAE, Complex Fractionated Atrial Electrograms; CPAP, Continuous Positive Airway Pressure; CRT-D, Cardiac Resynchronization Therapy with Defibrillator; DCC, Direct Current Cardioversion; DPP4i, Dipeptidyl Peptidase-4 inhibitor; EEG, Electroencephalography; EHRA, European Heart Rhythm Association; ESS, Epworth Sleepiness Scale; GAL, Galectin-3; GDF-15, Growth Differentiation Factor-15; GLP-1RA, Glucagon-Like Peptide-1 Receptor Agonist; GLP-1/GIP, Dual Glucagon-Like Peptide-1/Glucose-Dependent Insulinotropic Polypeptide receptor agonist; HbA1c, Glycated Haemoglobin; HFpEF, Heart Failure with preserved Ejection Fraction; HFrEF, Heart Failure with reduced Ejection Fraction; HSAT, Home Sleep Apnoea Testing; MET, Metabolic Equivalent of Task; MMP-9, Matrix Metalloproteinase-9; OSA, Obstructive Sleep Apnoea; PAP, Positive Airway Pressure; PSG, Polysomnography; PVI, Pulmonary Vein Isolation; PTX3, Pentraxin-3; RCT, Randomized Controlled Trial; ROS, Reactive Oxygen Species; VCAM1, Vascular Cell Adhesion Molecule-1; WHtR, Waist-to-Height Ratio; WHO, World Health Organization.
